# Supplementary material for: Universal consensus 3D segmentation of cells from 2D segmented stacks
Source: Nat Methods. 2025 Nov 11;22(11):2386–99. doi: 10.1038/s41592-025-02887-w (PMC12615268; doi:10.1038/s41592-025-02887-w)
Supplement: Supplementary file 1 — Supplementary Movie 1–13 (captions), Notes 1–5, Tables 1–5 (captions), Figs. 1–9 and References. [file 41592_2025_2887_MOESM1_ESM.pdf]

nature methods

Supplementary information https://doi.org/10.1038/s41592-025-02887-w

Universal consensus 3D segmentation of cells from 2D segmented stacks

In the format provided by the authors and unedited

Overview

Supplementary Movie Captions

Supplementary Note 1: Robust implementation of steps in general 2D-to-3D segmentation

Supplementary Note 2: Gradient smoothing and suppressed gradient descent are essential

Supplementary Note 3: Semi-automatic tuning of pretrained Cellpose models using local contrast

Supplementary Note 4: Applicability of u-Segment3D to any 2D segmentation method

Supplementary Note 5: Applying u-Segment3D to segment anisotropic 3D data

Supplementary Table Captions

Supplementary Figures: 1-9

Supplementary References

Supplementary Movie Captions

Supplementary Movie 1. u-Segment3D is a toolbox to integrate 2D segmentations from orthoviews into a single consensus 3D segmentation. Illustration of applying a 2D segmentation method to generate instance segmentations of cells within an image volume slice-by-slice from x-y, x-z, and y-z directions. This produces 3 separate 2D stacks which can be integrated using u-Segment3D into a single 3D segmentation. As visualized by exploding the end segmentation, this strategy can successfully extract every cell in dense cleared tissue volumes, without data training.

Supplementary Movie 2. Gradient descent dynamics of foreground cell coordinates using different 2D transforms. For each transform, we visualize i) the computed gradients color-coded by direction; ii) the advection of all foreground points (white dots) along the gradients using gradient descent; iii) the gradient descent trajectory (magenta) of equisampled boundary points; and iv) final reconstructed cells. Reconstructed cells are colored using the magma colorscheme from light-yellow to black. The color range is the same for all transforms and is set to be from zero (light-yellow) to 1.5 times the maximum cell id of reference cells (black). Perfectly reconstructed cells such as that using explicit ‘point’ source transforms, should be uniquely colored from light-yellow to purple. In contrast, oversegmentation causes an overabundance of blacks and undersegmentation an overabundance of yellows.

Supplementary Movie 3. Comparison of the spatial proximity clustering used by Cellpose 3D and u-Segment3D’s image-based connected component analysis on noisy cell tracking challenge datasets with non-optimized model parameters. For each example, the movie first rotates the rendering of the output 3D segmentation then flies through individual x-y slices where individual cells are delineated by black outlines. Each cell was assigned one of 16 colors from the ‘spectral’ colorscheme. The same cell in 3D render and 2D slices have the same color.

Supplementary Movie 4. Gradient descent dynamics of foreground cell coordinates under 3D gradients reconstructed from 2D gradients. Visualization of the propagated foreground points (magenta dots) under suppressed gradient descent with momentum for 200 iterations relative to initial positions (black dots) for the shapes and parameters in Suppl. Fig. 7b.

Supplementary Movie 5. Gradient descent dynamics during 3D reconstruction of ovules, lateral root primordia and vasculature from ideal 2D segmented stacks. Visualization of the propagated foreground points (magenta dots) under suppressed gradient descent with momentum for 250 iterations relative to initial positions for the exemplar images from ovules, lateral root primordia, and DeepVesselNet in Fig. 2d-f. Initial foreground positions are colored according to the unique reference cell they belong to. Rotation of the 3D rendering of reference (left) and reconstructed cells using centroid diffusion (middle) and Euclidean distance transforms (right). Whereas centroid point diffusion advects foreground points towards single point attractors, EDT advects foreground points towards the skeleton. When gradient decay, τ is sufficiently high as in DeepVesselNet, the behavior of both distance transforms are similar and points are minimally advected to separate individual vessel networks without fragmentation.

Supplementary Movie 6. Gradient descent dynamics of 3D reconstruction of lateral root primordial from only y-z, x-y and y-z and x-y, x-z and y-z Embedseg2D 2D segmented stacks. Visualization of the propagated foreground points (magenta dots) under suppressed gradient descent with momentum for 100 iterations relative to initial positions (black dots) for the exemplar test volume of lateral root primordia in Fig. 3g from using only y-z, both x-y and y-z and all three x-y, x-z and y-z Embedseg2D 2D segmented stacks (left-to-right). Rotation of the 3D rendering of the corresponding output u-Segment3D consensus 3D segmentations.

Supplementary Movie 7. Segmentation of a movie of thin MDA231 human breast carcinoma cells embedded in collagen from the 3D cell tracking challenge using u-Segment3D with stacks of 2D x-y segmentations only. Flythrough of x-y slices and 3D rotation of timepoint 0 of raw images and u-Segment3D segmentations. The x-y segmentations were generated using u-Segment3D's automatically determined optimal Cellpose 2D model parameters. Timelapse of raw image and u-Segment3D segmentations for all 15 timepoints.

Supplementary Movie 8. Segmentation of unwrapped surface cells of a Drosophila embryo over time using u-Segment3D to aggregate only 2D x-y segmentations only. Rendering of the surface extracted from binary embryo segmentation of each timepoint using u-Segment3D. Timelapse of the topographic volumes constructed by mapping a surface proximal volume into cartographic coordinates using u-Unwrap3D. Rotation and flythrough of the raw topographic volume at timepoint 25 and corresponding u-Segment3D segmentation. Timelapse of the topographic z-slice that best captures all surface cells and timelapse of the mid-slice y-z cross section (same as Extended Data Fig. 8m), with corresponding u-Segment3D segmentation.

Supplementary Movie 9. u-Segment3D postprocessing enables recovery of missing surface protrusions in the 3D segmentation of a HBEC cell aggregate. Rotating 3D renderings at each part of the workflow depicted in Fig. 4a.; (i) raw image, (ii) deconvolved image, (iii) consensus u-Segment3D segmentation of deconvolved image with Cellpose 2D outputs, (iv) segmentation following label diffusion and (v) segmentation following guided filtering postprocessing. Final volumes were meshed, exploded and rotated to visualize the whole cell segmentations this workflow extracts.

Supplementary Movie 10. Comparison of the segmentation of vessel sprouting in zebrafish without and with u-Segment3D postprocessing. Rotating 3D rendering of (i) raw image, (ii) consensus u-Segment3D binary segmentation of raw image using Cellpose 2D outputs and (iii) segmentation after label diffusion and guided filtering postprocessing.

Supplementary Movie 11. u-Segment3D segmentation of 43,779 cells in a $\approx 35\mu$m x 1.5mm x 1.5mm CYCIF multiplexed tissue section of metastatic melanoma. Flythrough of x-y slices of the entire volume and then in a zoomed-in region. Subsequent flythrough of x-z and y-z slices of the entire volume with simultaneous zoomed-in regions. This movie is compressed as MPEG-4 with the HEVC codec and can be viewed, for example, with the VLC media player.

Supplementary Movie 12. u-Segment3D segmentation enabled detection of weakly fluorescent lung micrometastases in cleared tissues. Rotations of 3D rendered input image with merged and separated TO-PRO-3 (nuclei, green) and luciferase-GFP (YUMM 1.7 cancer cells, magenta) channels; initial consensus u-Segment3D segmentations using Cellpose 2D outputs for nuclei and cancer cells; and cancer cells after filtering out spurious segmentations by luciferase-GFP intensity.

Supplementary Movie 13. u-Segment3D segmentation of heterogeneous cell structures in brain tissue labelled using coCATs. Rotating rendering of input image stack with consensus 3D segmentation of the larger pockets of extracellular space. Slice-by-slice flythrough of the segmentation, colored and overlaid using transparency onto raw x-y, x-z, and y-z slices. Individual pockets are additionally delineated by green outlines.

Supplementary Note 1: Robust implementation of steps in general 2D-to-3D segmentation

We developed u-Segment3D to robustly implement each step of the outlined general 2D-to-3D segmentation algorithm in the paper (Fig. 1f, Suppl. Movie 1). Specifically, this involved careful testing that the implementation of each step on representative exemplar images and shapes executed with the correct behavior, even on potentially pathological examples, which a robust framework should also handle. Furthermore, we introduce for each step, different computational options when applicable, and expose parameters to accommodate for the inevitable variations in the quality of 2D segmentations of real data.

First, as an object's medial-axis skeleton is not unique[1,2], u-Segment3D implements a choice of distance transforms to tune the segmentation with respect to computation speed, accuracy, and compatibility with 2D model outputs (Suppl. Fig. 1). These are categorized into two classes; 'explicit' (Suppl. Fig. 1b) or 'implicit' (Suppl. Fig. 1c). Explicit transforms define attractor coordinates that are incorporated as boundary conditions in the computation (Methods). This ensures gradients are zero inside the attractor and stable convergence via gradient descent (Suppl. Movie 2). u-Segment3D implements single 'point' and multi 'point set' source attractors. The single point is the internal medial centroid, whose placement is adjustable by percentile-based thresholding of the cells' Euclidean distance transform (EDT) (Suppl. Fig. 1d, Methods), extending the definition in Cellpose[3]. The point set is the 2D medial-axis skeleton of the cell shape computing by morphological thinning (Methods). To compute the distance transforms, u-Segment3D considers two different partial differential equations (PDEs); the Eikonal equation, which gives the geodesic solution; and the Poisson equation, which gives the heat diffusion solution, also used by Cellpose[3]. The solution of the Eikonal equation, offers piecewise smooth gradients but is efficiently computed by the fast marching method[4]. The Poisson equation offers smooth gradients, thereby reducing oversegmentation, but is slower to compute, being solved exactly using LU decomposition (Methods). Implicit transforms specify the medial skeleton as ridges. Convergence to the attractor is therefore unstable[1,5] (Suppl. Movie 2), however it is efficient, solving the PDEs without additional constraints. The Eikonal equation can then be solved using EDT, which is also an intermediary output of many 2D segmentation models[5,6]. Irrespective of the chosen distance transform, it is imperative for 2D-to-3D segmentation that the computational implementation guarantees a proper definition of the distance throughout the cell, regardless of cell shape. Iterative solutions of the PDE such as those implemented by Cellpose fail to fully diffuse in shapes (Suppl. Fig. 1e), particularly with elongated and tortuous substructures (Suppl. Fig. 1f), even with increased iterations. Consequently, u-Segment3D relies on exact solutions of the PDEs. Gradients remain properly defined and faithfully capture the shape (Suppl. Fig. 1f).

Second, u-Segment3D uses a content-based consensus averaging function, F, to fuse 2D image stacks, (Suppl. Fig. 2). 2D slice-by-slice segmentation may miss or under-/over-segment a cell across slices. Inspired by multiview image fusion[7,8], u-Segment3D fuses multiple image stacks using linear inverse local variance weighting (Suppl. Fig. 2a, Methods). Using EDT as an example, segmentation errors across slices cause non-continuity and high local spatial variance. Using inverse weighting, the pixel values with high-variance are down-weighted in the final fusion of images (Suppl. Fig. 2b). Increasing the local pixel neighborhood size enables correcting larger errors. A 1x1x1 pixel neighborhood, corresponds to a mean average fusion also used by Cellpose[3]. No error correction is applied when fusing. With a 5x5x5 pixel neighborhood, binary thresholding on the fused EDT perfectly recovers the foreground nuclei without artifacts (Suppl. Fig. 2c).

Third, 2D-to-3D segmentation requires accurate implementations of gradient descent in 2D and 3D, (Suppl. Fig. 3). For downstream spatial proximity clustering, gradient descent must propagate points of the same attractor together whilst retaining spatial compactness (Suppl. Fig. 3b). We verify our implementation using a synthetic 2D image of two objects, a circle within a doughnut (Suppl. Fig. 3a). Though simple, the object's gradient field is complex with features typical of more nuanced morphologies such as local sinks and separating flows of opposite orientation. Running 100 iterations, u-Segment3D propagates points stably converging towards the two point attractors (Suppl. Fig. 3c), enabling perfect reconstruction of the original objects. In contrast, Cellpose's implementation generates erroneous additional attractors: orphaning foreground points of the ring and an erroneous line attractor for the circle. Though minor in this example, inaccuracies in gradient descent compound with spatial clustering in the next step to detrimentally impact the final segmentation.

Fourth, to identify attractors in the gradient field u-Segment3D uses spatial proximity clustering of the propagated points by connected component analysis, (Suppl. Fig. 4). Too many or too few clusters directly

translate to over- and under- segmentation. With heterogeneous cell shapes, points do not converge to their attractors at equal speed. Running gradient descent to convergence for all cells is unnecessary and computationally expensive in 3D. Consequently, clustering must generalize to intermediate, irregular shapes represented by more uniform point densities. Density-based clustering methods such as the adaptive local histogram thresholding used by Cellpose[3] are unsuitable. These implicitly assume and find all local point-like hotspots as attractors. This leads to catastrophic failure, generating grossly fragmented cells. To overcome this, u-Segment3D exploits the fact that foreground coordinates are on an image grid (Suppl. Fig. 4a). The final floating-point advected coordinates are rasterized by integer flooring (step i). A count of the number of coordinates in each voxel is tabulated (step ii) and smoothed with a Gaussian filter of σ to build an approximate kernel density heatmap, ρ (step iii). ρ is sparse, enabling clusters to be identified using a global threshold, $mean(\rho) + k \cdot std(\rho)$ with tunable k. Connected component analysis then labels all spatially separated regions irrespective of shape and density with unique ids (step iv). The final segmentation is generated by indexing the final advected coordinates of foreground voxels in this label image, and transferring the labeling to the initial coordinates. ρ enables probabilistic cluster identification. By increasing σ u-Segment3D can 'fuzzy' link erroneously split clusters, equivalent to merging segmentations in the final 3D. We verified our implementation by reconstructing the 2D cell segmentation as we propagate foreground coordinates along the gradients of the geodesic centroid distance transform (Suppl. Fig. 4b i,ii). We correctly obtain the equivalent segmentation of applying connected component analysis to the foreground binary at iteration 0. As iterations increase, and attractors emerge, the segmented cells converge on the true number (Suppl. Fig. 4b iii, top). Correspondingly, segmentation quality, measured by the intersection-over-union (IoU) and F1 score, increases to 1 (Suppl. Fig. 4b iii, bottom). These observations also translate to elongated, touching cells (Suppl. Fig. 4c). Moreover, only our clustering recovers the number of clusters present in the final coordinates propagated by either Cellpose or u-Segment3D's gradient descent for the synthetic image of a circle within a doughnut (Suppl. Fig. 3d, 4d). Cellpose's clustering breaks up what should be single clusters (Suppl. Fig. 3d). This negatively impacts Cellpose's 3D segmentation performance, particularly for low signal-to-noise ratio or out-of-distribution cells (Suppl. Fig. 4e,f). Whereas Cellpose's gradient descent and clustering grossly oversegments and fractures individual cells, u-Segment3D's gradient descent and connected component clustering recovers complete cell segmentations when applied to reparse the same predicted foreground binary and 3D gradients (Suppl. Fig. 4e,f, Suppl. Movie 3).

To maximize the utility of pretrained 2D models, u-Segment3D further implements preprocessing and postprocessing modules. The image to segment may not reflect the quality, acquisition, noise distribution and modality of the training dataset a segmentation model was trained on. Preprocessing can help transform input images to improve performance in the 2D segmentation of orthoview slices[9,10]. While dataset- and model-specific, the following order of processing steps has proven broadly applicable: intensity normalization, none or any combination of denoising, deconvolution and ridge feature enhancement, and uneven illumination correction with optional gamma correction (Methods). Postprocessing steps are implemented; to filter out implausible segmentations by object size and consistency with the reconstructed 3D gradients (as in Cellpose[3]), to refine segmentations by spatial-connectivity aware label diffusion to better adhere to cell boundaries within a guide image; and guided filtering to recover missing or intricate subcellular details. None of these postprocessing procedures require further 3D training (Methods).

Supplementary Note 2: Gradient smoothing and suppressed gradient descent are essential

Smoothing of reconstructed gradients and suppressed gradient descent are essential for 1D-to-2D segmentation

To understand how the different components of the 2D-to-3D algorithm may impact 3D segmentation, we first empirically investigated 1D-to-2D reconstruction of single cell shapes from the Cellpose[3] and Omnipose[5] training datasets, which can be intuitively visualized (Suppl. Fig. 5). We first examined the approximation of 2D gradients using 1D gradients (Suppl. Fig. 5a,b).

To compute 1D gradients, each spatially disconnected 1D region in a 1D slice was treated as a unique 'cell'. For each 'cell', we computed the distance of its foreground points to the cells' centroid, computed the 1D gradient using central differences and unit length normalized the vectors. The 2D gradients are reconstructed by stacking the 1D gradients computed from x- and y- directions, and smoothed by applying an isotropic 2D Gaussian filter, width σ to x- and y- components. Gradient descent was iteratively performed to compute the trajectory, and the 2D segmentation was obtained by applying the image-based connected component analysis of u-Segment3D to the final advected 2D coordinates.

Across single cells representing spherical, convex, branched and vessel morphologies, we found that Gaussian filtering of the reconstructed 2D gradients was essential to recover the original 2D shapes (Suppl. Fig. 5b). Without smoothing, the 2D gradients specified by the raw 1D x-, y-gradients (Methods), do not capture the 2D context and specify a single fixed-point attractor per cell. Only by smoothing reconstructed 2D gradients with increasing σ, trajectories were regularized to converge towards single point attractors. Smoothing effectively conformalizes the cell shape, shifting its 2D centroid towards the centroid of its convex hull. Unfortunately, for concave shapes, the new attractor may lie outside the cell. To examine the implications of this with crowded cells, we performed the same 1D-to-2D reconstruction considering full images with densely packed 2D cells. For a Cellpose example (90 cells), the reconstructed 2D gradients smoothed by $\sigma = 1$ contains more than one attractor per cell (Suppl. Fig. 6a). Consequently, after 50 iterations of gradient descent we oversegment (143 cells). Nevertheless, the shape reconstruction is good ($F1 = 0.77$, IoU=0.91). Moreover, for fragmented cells, the number of fragments per cell was low, and were largely small fragments. Increasing σ regularizes the reconstructed gradients. For $\sigma = 5$, segmentation (93 cells, F1=0.94, IoU=0.93) is on-par with ideal 2D gradients (93 cells, F1=0.98, IoU=1.00). Beyond $\sigma > 5$, gradients interact across neighboring cells, causing undersegmentation, as well as decreased IoU and F1 performance (Suppl. Fig. 6a iv). For long and thin tubular structures in the Omnipose example (86 cells), increasing σ shifts attractor centroids into neighboring cells. Thus increasing σ improved F1 but also decreased IoU, with an optimal balance at $\sigma = 3$ (F1=0.49, IoU=0.60) (Suppl. Fig. 6b iv). Due to the sensitivity to cell size and shape, tuning σ is impractical. The aim of gradient descent is to separate adjacent cells by clustering points towards their medial-axis skeleton. Iterations beyond this point are unnecessary. Depending on the distance transform (Suppl. Movie 2) they can cause fragmented clusters and thus oversegmentation. To prevent excessive iterations without changing the preset iteration maximum, we define a variable stepsize, $\eta = \frac{1}{1+\tau \cdot t}$, decaying for higher iterations, where t denotes the iteration and τ an adjustable decay rate[5]. Applying the suppressed gradient descent[5] with $\sigma = 1$ perfectly reconstructed the 2D shapes (86 cells, F1=1.00, IoU=1.00) (Suppl. Fig. 6c).

Translating gradient smoothing and suppressed gradient descent to the case of 2D-to-3D segmentation

To test if insights from the 1D-to-2D segmentation translate to 2D-to-3D segmentation we conducted the analogous reconstruction experiment for single 3D cells (Suppl. Fig. 7a). These cells were segmented from high-resolution microscopy images as surface meshes by u-Shape3D[11], then voxelized to binary volumes using u-Unwrap3D[12]. Similar to the 1D-to-2D case, the 2D geodesic centroid distance transform (Methods) was computed slice-by-slice in orthogonal x-y, x-z, y-z stacks, treating spatially contiguous 2D regions as unique 'cells'. The 3D gradients were then reconstructed by averaging (F with 1x1x1 pixel neighborhood). Different, however, is that we additionally incorporate momentum into the suppressed gradient descent to

expedite convergence (Methods). 3D cells were selected to represent a spectrum of morphologies ranging from pseudo-spherical to pseudo-convex and branched, and with different types of surface protrusions (Suppl. Fig. 7b). Applying suppressed gradient descent ($\tau = 0.1$) for 200 iterations, we found similar results as 1D-to-2D, with 3D cell examples of pseudo-spherical, pseudo-convex and branched morphologies (Suppl. Fig. 7b, Suppl. Movie 4). Gaussian smoothing again aids regularization, and increasing σ ensures convergence to a single cell, even for a highly branched cell with filopodia ($\sigma = 15$). The same cell was fragmented into several regions at branch junctions at lower $\sigma = 1$. However, we can also fix $\sigma = 1$, and increase $\tau = 0.5$ to still recover perfect construction of the branched cell. As large σ risks smoothing across cells, we recommend primarily tuning τ.

In summary, these experiments underline the necessity of Gaussian filtering to smooth the reconstructed 3D gradients, and the use of a suppressed gradient descent. These are among the algorithmic key differences to Cellpose that allow u-Segment3D to segment cells of heterogeneous morphologies.

Supplementary Note 3: Semi-automatic tuning of pretrained Cellpose models using local contrast

When working with pretrained Cellpose 2D models two model parameters crucially influenced performance: (i) the diameter defining the expected cell object size and (ii) the cell probability threshold to determine the foreground binary. Cellpose 2D models enable 'optimal' diameter prediction using a pretrained regression model. However, this assumes one size fits all. An image can contain objects of different scales, e.g. cell body vs cell nuclei, cells within an embryo vs the overall embryo shape. Moreover, a trained model cannot guarantee generalization to out-of-sample datasets or consistency across sequential 2D slices. When we examined cell probability and gradients on cross-sections of the LRP dataset predicted by Cellpose, we found seemingly similar results over a broad range of 'diameter' settings (Suppl. Fig.8a-c). To set the 'diameter' objectively without training, we developed an alternative tuning method based on examining the model's self-confidence. u-Segment3D runs Cellpose over a test diameter range to compute a 'contrast score' per diameter using the local pixel variance within the predicted gradients and cell probability (Suppl. Fig. 9a, Methods). The resulting contrast function uncovers all salient object scales as local maxima, serving as a tuning guide for diameter. Cellpose models are trained using a mean diameter of 30 pixels and documented to perform best for diameter=15-45 pixels. Therefore, based on the peak of the contrast function, images are resized in preprocessing (Suppl. Table 1) to match Cellpose's optimal operating diameter range. For automated operation, u-Segment3D selects the diameter with maximum contrast as optimal. If multiple peaks are present, the optimal diameter can be biased to favor alternative maxima by adjusting the size of the considered pixel neighborhood used to compute the contrast score (Suppl. Fig. 9, Suppl. Table 1) or by constraining the diameter range. In 3D, the cross-sectional appearance of an object often has different aspect ratios and size, even if the image is resized to isotropic voxels. We apply our tuning to set the optimal diameter in each of the x-y, x-z, and y-z views using a representative 2D slice (Methods). As validation, the Cellpose predicted diameter coincides with the predicted maxima of our method on the same 2D image (Suppl. Fig. 10a-c). Moreover, the direct method 3D segmentation using our method ($\mathrm{AP}_{0.5}$=0.28) is comparable to using Cellpose's method ($\mathrm{AP}_{0.5}$=0.23), if not better (Suppl. Fig. 10d).

For thresholding the cell probability u-Segment3D uses multi-threshold Otsu to statistically determine a finite choice of thresholds (Methods). Cellpose does not provide automated means. We can use flooring to round thresholds to the nearest decimal point or select a lower threshold to strike a balance between segmentation accuracy and ensuring contiguous foreground regions for gradient descent. This works excellently for both 2D Cellpose and for 3D reconstructed cell probabilities. Given the problems we found with Cellpose's gradient descent (Suppl. Fig. 3), and density-based spatial clustering (Suppl. Fig. 4), in the paper we always use u-Segment3D's equivalent to generate segmentations. Unless otherwise stated, we refer to Cellpose 2D outputs as being its predicted gradients and cell probability, segmentation to the cell masks after applying to Cellpose 2D outputs u-Segment3D's statistically-informed binary thresholding, suppressed gradient descent with momentum and connected component analysis.

Supplementary Note 4: Applicability of u-Segment3D to any 2D segmentation method

2D segmentation models either (i) already predict a suitable distance transform or 2D gradients (c.f. Suppl. Fig. 2, Methods), e.g. Cellpose[3], or (ii) provide 2D instance segmentation masks from which distance transforms may be computed. u-Segment3D accounts for both cases (Extended Data Fig. 3a). In the former, predicted 2D gradients are directly used to generate the 3D segmentation (the direct method). In the latter, users choose a 2D distance transform to compute the necessary 2D gradients from the 2D segmentation masks, (the indirect method). We demonstrate both methods using pretrained generalist Cellpose models. Unlike the ideal 2D projections derived from given 3D shapes, the morphology of the reconstructed 3D foreground binary is an additional determinant of the overall performance. If the foreground does not provide a contiguous path for gradient descent, the resulting segmentation will be fragmented, even with correct gradients.

Using our tuning and parsing of Cellpose, we compared the direct and indirect method of u-Segment3D on 9/11 datasets (see Suppl. Table. 4 for parameter details). The zebrafish macrophage dataset was excluded, as labels were derived from u-Segment3D; and VesselMNIST3D, which only contains binary masks. We also considered two pretrained Cellpose models, 'cyto' and 'cyto2', both generalist models but trained on different datasets, to assess how different 2D models translate to different 3D segmentation performance. The direct result was assessed relative to Cellpose 3D and native 3D segmentation baselines. For the latter, we used an unsupervised 3D watershed as the lower bound, and dataset-specific native 3D segmentation model as the upper bound (Methods). The indirect result was compared to the stitching methods, CellComposer and CellStitch. To minimize data leakage, we applied models to only the validation or test splits when available.

On Ovules validation split (n=2,840 cells, m=2 images, Extended Data Fig. 3b i), we found excellent performance with both the 'cyto' and 'cyto2' models and the direct method, surpassing the watershed and comparable to the specialized native pretrained PlantSeg3D[13] models (Fig. 3b ii, $\mathrm{AP}_{50} \approx 0.80$ for both). This was expected as the cells are convex and delineated by high-contrast edges. This is also evidenced by good performance of running Cellpose 3D on the same preprocessed input. Cellpose 3D has no automatic diameter tuning and uses only one diameter across all views. To perform a fair comparison without modifying its source code, we accounted for the oversegmentation tendency of Cellpose 3D and used the maximum of u-Segment3D inferred diameters. We also ran Cellpose 3D twice, the first to obtain 3D cell probabilities to compute the equivalent Otsu thresholds and the second to obtain final segmentations using the same postprocessing parameters (Methods). As expected from algorithm design, for the same model, u-Segment3D consistently outperforms Cellpose 3D. Most impressively, u-Segment3D even boosted the 'cyto' model from $AP_{0.5} = 0.7$ to $AP_{0.5} = 0.8$ to be on par with 'cyto2'. The same was true for the test split (n=10,328 cells, m=7 images, Extended Data Fig. 4). Again u-Segment3D raised the average precision of 'cyto' from $AP_{0.5} = 0.65$ to $AP_{0.5} = 0.7$. Compared to the best 3D reconstruction with ideal 2D segmentations (black line, circle marker, ($\mathrm{AP}_{50} = 1.0$), however, there remains a noticeable gap of 0.2. Interestingly, the indirect method with either the geodesic (magenta colored) or diffusion (cyan colored) distance transforms for both models was better quantitatively than the direct method (Fig. 3b iii, $\mathrm{AP}_{50} > 0.80$). This is likely due to better cell boundary delineation from aggregating on the hard-thresholded 2D segmentations. However, the total number of cells predicted decreased (reference=1686, direct=1697, indirect=1529). In contrast, CellComposer and CellStitch are severely impeded by non-ideal Cellpose 2D segmentations. The stitched 3D segmentation was worse than 3D watershed ($\mathrm{AP}_{0.5}$ = 0.2-0.3), a > 0.5 decrease from using ideal 2D slices (Fig. 2d). Lastly, we tested whether the 2D orthoslices of the direct 3D segmentation (black lines) retain the accuracy of 2D segmentations. We compared the native 2D slice-by-slice segmentations in x-y, x-z, and y-z views (magenta lines) to the corresponding cross-sections of the 2D-3D consensus segmentation by u-Segment3D as translated by the direct method (Extended Data Fig. 3b iv). Not only does u-Segment3D preserve the 2D segmentation but even improved it in x-y view for both models ($\mathrm{AP}_{0.5}$ = 0.45 to 0.55). Meanwhile, CellComposer and CellStitch underperform with a strong bias against the x-z and y-z views. This demonstrates the true, consensus integration of complementary information from orthogonal predictions by u-Segment3D.

The segmentation of the LRP data set is more challenging as it contains a mixture of both compact and elongated/branching cells with weakly-defined edges (Extended Data Fig. 3c). Unsurprisingly, direct u-Segment3D segmentation with both models on the validation split was substantially lower ($\mathrm{AP}_{50} \approx 0.30$ for both) than that from ideal 2D segmentations ($\mathrm{AP}_{50} \approx 0.90$). Nevertheless, u-Segment3D significantly

outperforms Cellpose 3D and watershed in both the validation and test splits (Extended Data Fig. 3,4, $\mathrm{AP}_{50} \approx 0.05$ for both; improving Cellpose 3D cyto ($\mathrm{AP}_{50} = 0.18$ to 0.37) and cyto2 ($\mathrm{AP}_{50} = 0.19$ to 0.40)). As with Ovules, AP_{50} was lower than PlantSeg3D model. However, this AP_{50} with pretrained Cellpose models with u-Segment3D was comparable to an Omnipose model (plant-omni) trained natively in 3D specifically on LRP[5]. We asked whether a specialist Cellpose 2D model (plant-cp) pretrained on 2D slices of LRP[5] would improve 3D performance. Performing a like-for-like evaluation (Methods), we find 2D plant-cp using indirect u-Segment3D and any distance transform outperformed 3D-trained plant-omni and generalist Cellpose 2D models for both validation ($\mathrm{AP}_{0.5}$=0.50, Extended Data Fig. 4c) and test ($\mathrm{AP}_{0.5}$=0.50-0.56) splits, comparable to PlantSeg3D (Extended Data Fig. 3c, Extended Data Fig. 4c,d). Unexpectedly, plant-omni and plant-cp (using Cellpose 3D) were only as good as pretrained cyto2 and direct u-Segment3D in both splits. Closer inspection revealed that though plant-omni looks excellent in 3D renderings, segmentations were incomplete when viewed cross-sectionally. Additionally, plant-omni oversegments despite additional size filtering (Methods). These results highlight the robustness of u-Segment3D and verify that the framework translates improved 2D segmentation models into better 3D segmentations, on-par or better than natively 3D trained models. Again, direct and indirect u-Segment3D segmentations were comparable in AP_{50}, and outperformed CellStitch and CellComposer equivalents. Indirect segmentations were better in IoU, with a slightly slower drop-off (Extended Data Fig. 3c iii). Also for LRP, u-Segment3D exploits complementary information from all orthogonal views: the 2D slice segmentations of the consensus 3D segmentation is marginally worse than Cellpose 2D segmentations in y-z AP curve, but improves in x-y and x-z AP curve performance for both 'cyto' and 'cyto2' models (Extended Data Fig. 3c iv). In contrast, despite the y-z view being most convex-shaped and having the best 2D segmentation, it was worst after stitching using CellStitch and CellComposer.

DeepVesselNet, comprised of thin, complex vasculature networks represents the biggest challenge for 2D-to-3D segmentation (Extended Data Fig. 3d). Both pretrained Cellpose models predict segmentations uniformly larger than the actual vessel radii in 2D slices. Hence, we additionally uniformly eroded consensus 3D segmentations to obtain the final segmentation (Suppl. Table 4). Nevertheless, there was a clear difference between the two models. Using direct segmentation, 'cyto' ($\mathrm{AP}_{0.5} = 0.5$, IoU drop-off$\approx$0.75) outperforms 'cyto2' ($\mathrm{AP}_{0.5} = 0.4$, IoU drop-off$\approx$0.65) (Fig. 3d ii). Without suppressed gradient descent, Cellpose 3D grossly oversegments ($\mathrm{AP}_{0.5} = 0$). Again, direct and indirect u-Segment3D segmentations were on-par in AP_{50}. However, the indirect method is superior in IoU, with drop-off extending to 0.95 with similar AP curves across all distance transforms (Extended Data Fig. 3d iii). As with ideal slices (Fig. 2f), CellComposer and CellStitch oversegment ($\mathrm{AP}_{0.5} = 0$). Comparing 2D segmentation performance, the direct 3D aggregated cyto outperforms individual 2D segmentations in $\mathrm{AP}_{0.5}$ but exhibits faster IoU drop-off (Extended Data Fig. 3d iv). The direct aggregated cyto2 was significantly worse than its 2D counterpart. This is likely due to the 3D erosion postprocessing removing too many small 2D segmentations in slices. Since the background appeared homogeneous in this dataset, we additionally tested 2D binary Otsu thresholding and connected component analysis instance masks. This yielded the highest $\mathrm{AP}_{0.5}$ 2D segmentations in all orthogonal views (Extended Data Fig. 3d iv, magenta circle lines). Applying u-Segment3D, we achieve the highest $\mathrm{AP}_{0.5}$ 3D segmentation ($\mathrm{AP}_{0.5} \approx 0.6$) with a 2D method (Extended Data Fig. 3d iii, turquoise circle line) but this was lower than native 3D binary Otsu thresholding and connected component analysis, ($\mathrm{AP}_{0.5} \approx 0.8$, Extended Data Fig. 3d (ii), green circle line). Impressively, the consensus u-Segment3D (Extended Data Fig. 3d iv, turquoise circle line) significantly improves upon the original 2D Otsu segmentations (Extended Data Fig. 3d iv, turquoise circle line), unlike CellComposer and CellStitch. Therefore, we attribute the discrepancy in performance to discretization, whereby three orthoviews are insufficient to fully capture the 3D branching directions.

Altogether, these analyses demonstrate the robust implementation and universal applicability of u-Segment3D to real datasets of different super-morphotypes. We also showed how u-Segment3D can be applied to any 2D segmentation method generating pixel-wise distance transforms and gradients or instance masks using the direct or indirect methods, respectively, with similar $\mathrm{AP}_{0.5}$. Thus, we applied only the direct method on remainder EmbedSeg[14] datasets with 3D watershed and specialist-trained EmbedSeg3D native 3D segmentation baselines (Extended Data Fig. 5, Methods). Except for Arabidopsis (CAM) (best $\mathrm{AP}_{0.5} = 0.4$), which had low image quality and densely-packed cells, all others had one pretrained Cellpose model outperforming watershed and $\mathrm{AP}_{0.5} \geq 0.6$. Mouse organoids and skull nuclei matched EmbedSeg3D. For mouse organoids, pretrained cyto2 with u-Segment3D ($\mathrm{AP}_{0.5}$ =0.93) nearly matched the ideal 2D segmentation ($\mathrm{AP}_{0.5}$=1.0).

Supplementary Note 5: Applying u-Segment3D to segment anisotropic 3D data

Due to the microscope or culture conditions, 3D images cannot always be acquired isotropically or be interpolated to be near-isotropic with an image quality similar in x-y, x-z, and y-z orthoviews. In these cases, application of 2D models, trained solely on the equivalent of in-focus 'x-y' slices, to x-z and y-z views may yield worse 3D segmentations. To accommodate this, u-Segment3D can use only x-y slices. u-Segment3D then operates similarly to slice-by-slice stitching method but retains the advantage of adjusting parameters such as smoothing to interpolate missing segmentations (Suppl. Table 1). Looking top-to-bottom through an epidermal organoid culture[15] (Methods), cells are flat and elongated in the suprabasal layers transitioning to cuboidal in the basal layer (Extended Data Fig. 8a). Even when interpolated to isotropic voxels, suprabasal cells remain flat, and stretched in appearance (Extended Data Fig. 8b). We applied pretrained Cellpose 2D to segment only x-y slices, finding an optimal diameter per slice, then translated the 2D segmentations to 3D. We compared the result from 2D segmentations using Cellpose (Extended Data Fig. 8c,d) and u-Segment3D (Extended Data Fig. 8e,f) generated optimal diameters. Qualitatively, both looked similar. Without ground-truth, and ambiguity in manual labeling without a nuclear marker, we assessed the segmentation consistency between consecutive x-y slices, slice i and slice $i+1$ with $\mathrm{AP}_{0.5}$. This revealed $\mathrm{AP}_{0.5}$ variation correlated with shape, with a systematic drop in $\mathrm{AP}_{0.5}$ as from squamous to cuboidal cell morphologies. Overall, u-Segment3D's method appears more stable, with a higher mean $\mathrm{AP}_{0.5} = 0.59$. We plotted the predicted mean cell diameter per slice (green line) with the measured cell diameter of the resultant segmentation (black line) for each method (Extended Data Fig. 8g,h). Whilst Cellpose better predicts the absolute mean diameter per slice, its correlation across x-y slices was moderate (Pearson's *R* = 0.47). In contrast, u-Segment3D's contrast-score method exhibits a strong correlation (Pearson's *R* = 0.89). This consistency likely translated to the improved slice-to-slice $\mathrm{AP}_{0.5}$.

A second example is a video of MDA231 human breast carcinoma cells embedded in a collagen matrix from the single cell tracking challenge[16] (Extended Data Fig. 8j). These cells have small area, thin, protrusive morphologies, and were imaged with a noisy background. The 3D image has only 30 z slices, each cell spanning <5 slices. Again, applying pretrained Cellpose with contrast-score diameter determination on x-y slices only, we generated consistent 3D cell segmentations. Visual inspection confirmed the same cell was consistently segmented across slices. Applying to every timepoint, we obtained consistent segmentation of cells across time (Suppl. Movie 7).

Finally, we also segmented cells on the surface of a developing drosophila embryo, a second data set from the single-cell tracking challenge[16]. Due to the curved embryo surface, cell dynamics are better visualized in cartographic surface projections[17]. We segmented the embryo surface with u-Segment3D (Methods) and applied the u-Unwrap3D[12] pipeline to define a cartographic projection of the surface proximal subvolume (Extended Data Fig. 8k). We then applied u-Segment3D to this computationally flattened tissue section to extract individual 3D cells. Again, due to the relatively shallow depth of the section we used x-y slices only. Despite the distortions introduced by the tissue projection, u-Segment3D still produced consistent 3D cell segmentations (Extended Data Fig. 8l). This enabled us to visualize the migration of cells toward the ventral midline (black arrows) from the side in relation to cells underneath the embryo surface (Extended Data Fig. 8m, Suppl. Movie 8).

Supplementary Table Captions

Supplementary Table 1. Table of all parameters in u-Segment3D code with explanation and tuning guidance. The variable names correspond to that used in the GitHub source code.

Supplementary Table 2. Summary of datasets used for validation of u-Segment3D. Unless otherwise noted, all images were first resampled to isotropic voxel resolution by appropriate downsampling of x-y slices and then resized isotropically by the indicated rescaling factor.

Supplementary Table 3. u-Segment3D settings and parameters for testing the reconstruction of 3D segmentation from ideal 2D segmentations with public datasets.

Supplementary Table 4. u-Segment3D settings and parameters for consensus 3D segmentation from the output of pretrained Cellpose 2D predictions with public datasets. The model inversion parameter is for Cellpose2 code. Model inversion is unnecessary for Cellpose3 code.

Supplementary Table 5. Cellpose2D and u-Segment3D settings and parameters for consensus 3D segmentation on additional demonstration datasets. The model inversion parameter is for Cellpose2 code. Model inversion is unnecessary for Cellpose3 code.

Supplementary Figures: 1-9

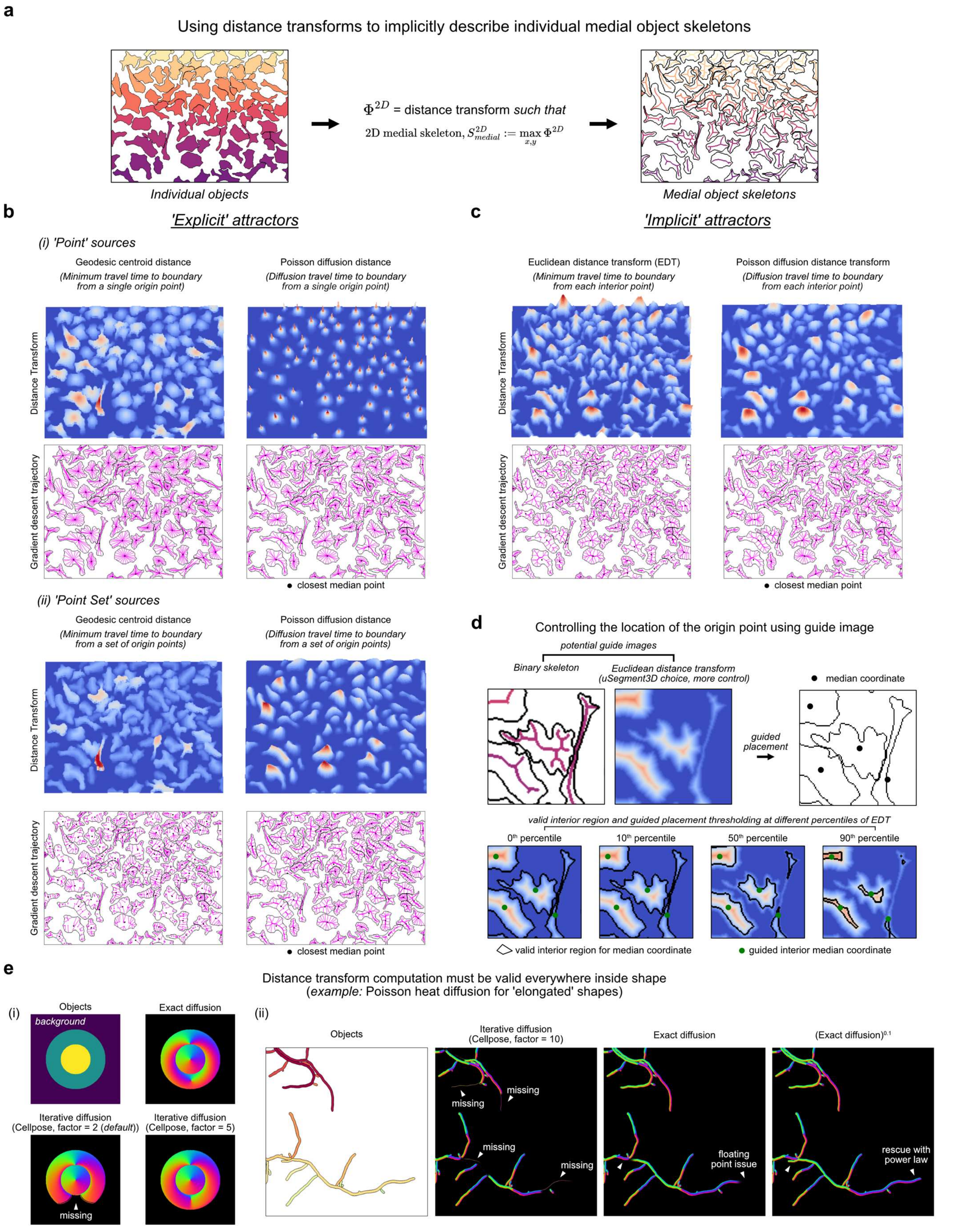

Supplementary Figure 1. 2D distance transforms for consensus 2D-to-3D segmentation in u-Segment3D. a) Necessity of 2D distance transforms to sample the 2D medial skeleton of individual shapes. **b)** Example of first-order (geodesic, left) and second-order (diffusion, right) distance transforms with 'explicit' attractors implemented in u-Segment3D. The limit of a gradient descent is explicitly defined as either (i) a single point-source on the 2D medial skeleton or (ii) a set of points along the 2D medial skeleton. **c)** Example

of first-order (geodesic, left) and second-order (diffusion, right) distance transforms implemented in u-Segment3D with ‘implicit’, skeletal-based attractors. Contrary to transforms with explicit attractors, here the gradient descent may not necessarily converge to the limit. For each example in **b), c)**, distance transforms are represented as a topographic relative heat map, colored blue (lowest) to red (highest) (top) by distance from the cell boundary, and by trajectories (magenta) of equi-sampled boundary points converging to the attractor under gradient descent (bottom). Black point = closest internal point to the median shape coordinate. **d)** Illustration of using percentile-based thresholding of the Euclidean distance transform of individual cells as a soft constraint to find the medial centroid 2D coordinate for convex and concave shapes used in computing the 2D point-source distance transforms in c). **e)** Unit-normalized 2D gradients for circle in doughnut synthetic shape. **f)** Unit-normalized 2D gradients for (i) elongated touching bacterial shapes using (ii) the simulated diffusion approach as implemented in Cellpose versus (iii) the exact solution of diffusion equation with boundary conditions as implemented in u-Segment3D (see Methods). Using an exact solver, computed gradients remain defined and are never zero, even in very long structures. However, due to floating-point precision, the gradient magnitude far from the source is no longer unit length, affecting the convergence of gradient descent. Thanks to the non-zero gradient magnitudes, u-Segment3D can rectify this situation, by (iv) raising the distance transform by an exponent p, Φ^p before computing the gradient. 2D gradients in panels (ii-iv) are colored according to direction. The number of simulated diffusion steps in Cellpose varies per shape and equals the factor multiplied by the number of pixels covered by the shape.

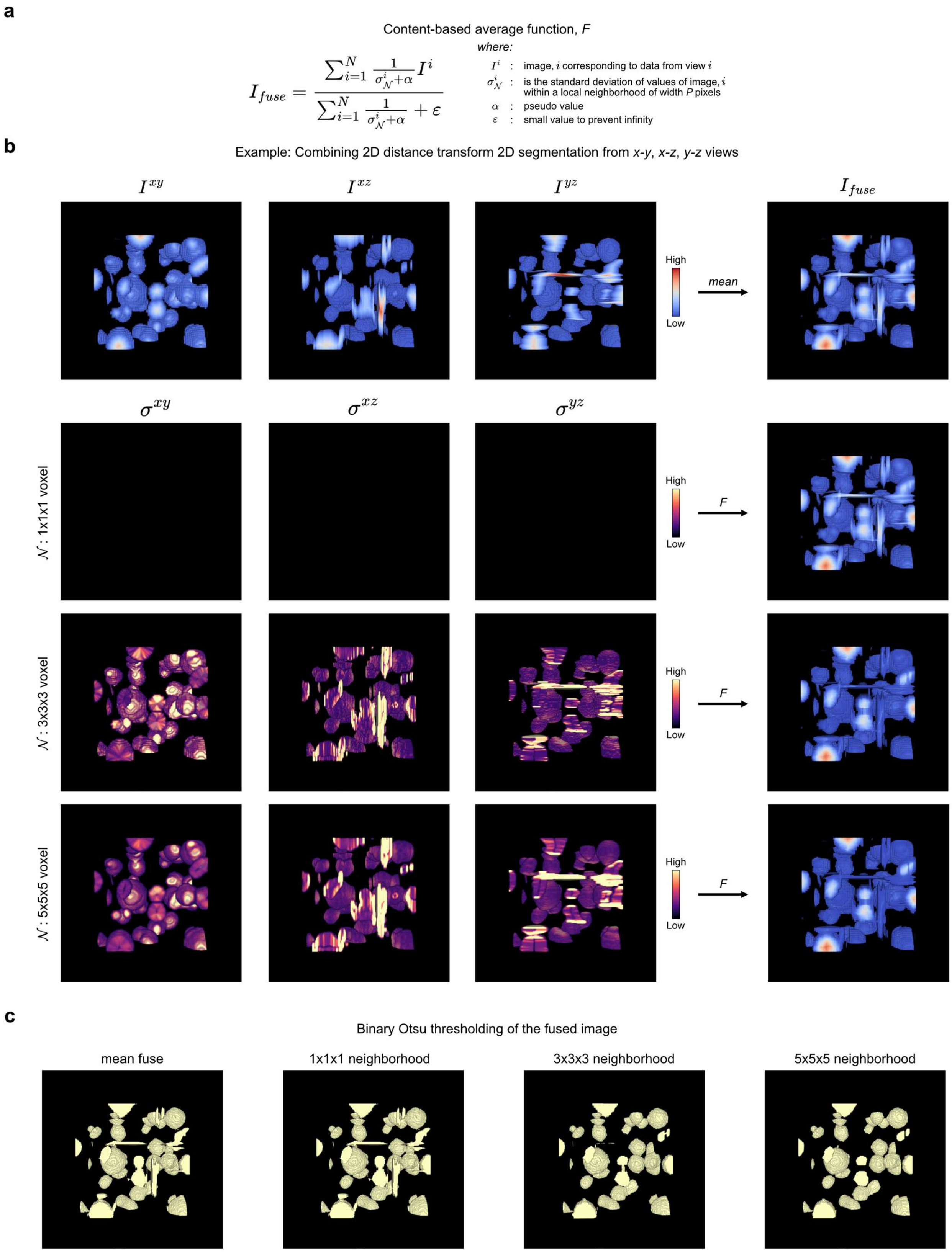

Suplementary Figure 2. Content-based average function for combining data from multiple views for consensus 2D-to-3D segmentation. a) Mathematical definition of the content-based average function as the inverse local variance weighted mean of input image values in an isotropic neighborhood of width *P* pixels. **b)** Example of applying the average function defined by a) to fuse the stacked 2D distance transforms after 2D slice-by-slice segmentation of a volume of spherical cells from x-y, x-z and y-z views. 1st row: Left-to-right, the Euclidean distance transform colored blue (low) to red (high) from the three orthoviews and fused distance transform using pixelwise mean. 2nd row: Left-to-right, the per-pixel local variance weight image σ for each orthoview for a neighbourhood of width P = 1 pixel and resultant fused distance transform using content-

based averaging. 3rd row: Left-to-right, the per-pixel local variance weight image σ for each orthoview for a neighborhood of width P = 3 pixel and resultant fused distance transform using content-based averaging. 4th row: Left-to-right, the per-pixel local variance weight image σ for each orthoview for a neighborhood of width P = 5 pixel and resultant fused distance transform using content-based averaging. **c)** Result of applying binary Otsu thresholding on the fused distance transform based on pixelwise mean, and content-based averaging using a neighborhood of width P = 1, 3, 5 pixels from left-to-right.

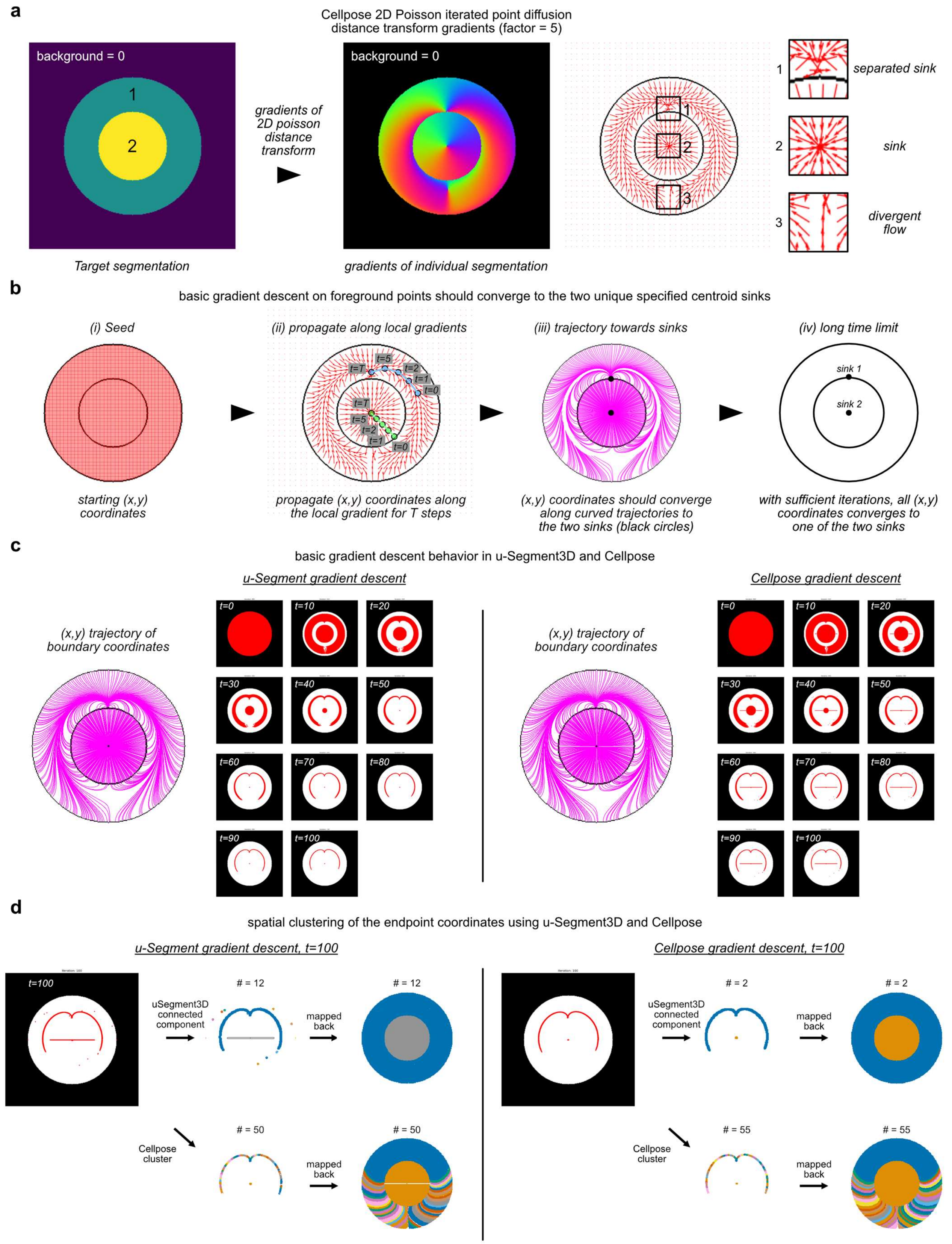

Supplementary Figure 3. Illustration of 2D Gradient descent for reversible 2D shape erosion. a) Cellpose computed 2D gradients for a synthetic shape of a doughnut (labelled 1) surrounding a circle (labelled 2) (left) with the 2D gradients visualized as red arrows (right) and zoomed in at three regions with different types of flow behavior. The number of simulated steps equals the factor = 5 multiplied by the number of pixels occupied per shape. **b)** Schematic of the expected behavior when gradient descent is iteratively applied to propagate the initial foreground (x,y) coordinates with the 2D gradients, the limit being convergence to the 2 black centroids. **c)** Observed point trajectories (magenta lines) and snapshots of coordinate positions

(red points in images) running gradient descent in u-Segment3D (left) vs Cellpose (right) for 100 iterations. Red features indicate the point aggregates that emerge after the indicated number of gradient descent iterations. **d)** Recovered cell shapes based on applying u-Segment3D (top) or Cellpose (bottom) spatial proximity clustering on the final coordinate positions after 100 iterations of u-Segment3D (left) or Cellpose (right) gradient descent. The maps demonstrate the critical role optimal numerical implementation of gradient descent plays in avoiding generating severely over-segmented objects.

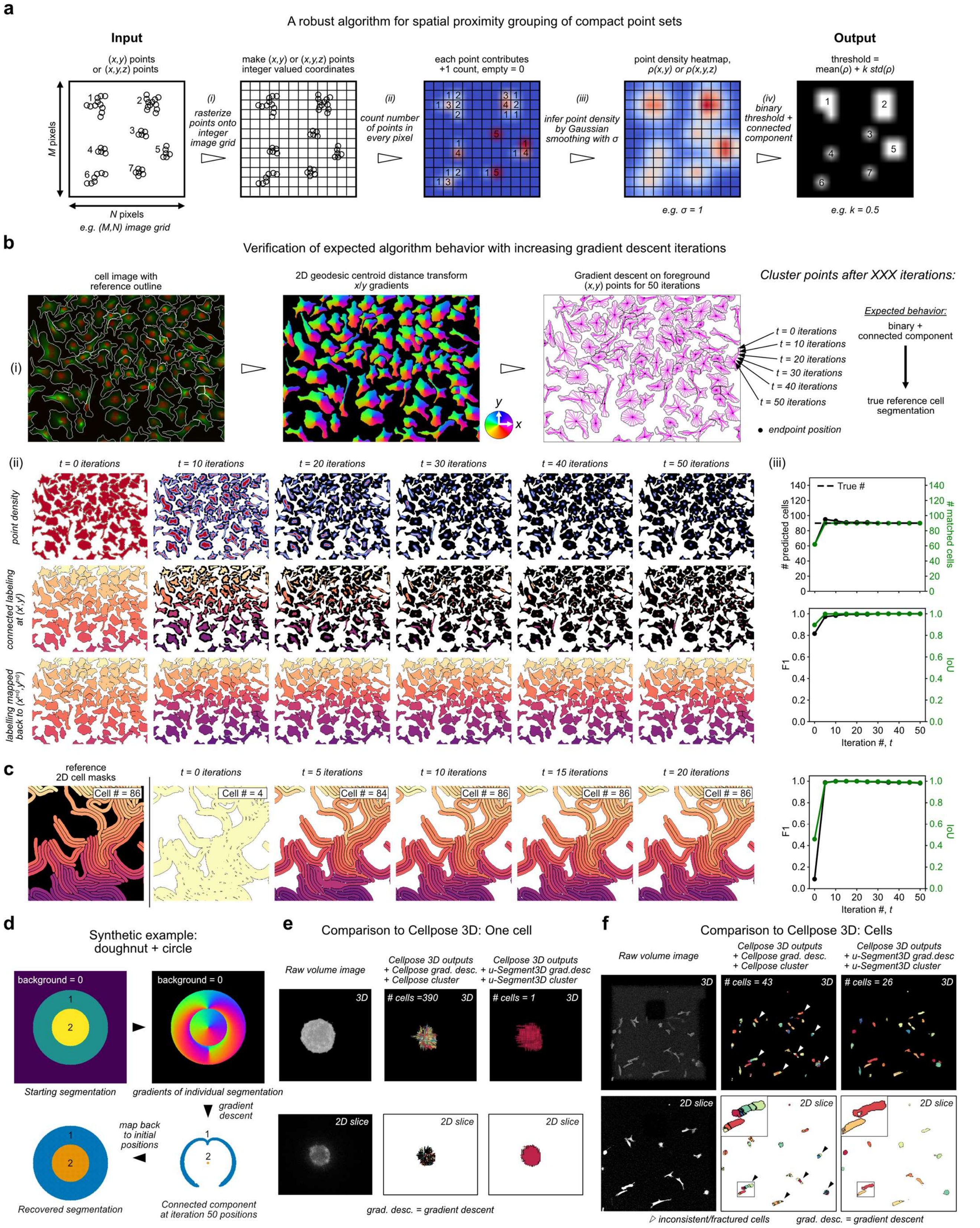

Supplementary Figure 4. Using image-based spatial connected component analysis to robustly identify distinct spatially compact point sets. a) 2D Illustration of the image-based connected components spatial clustering approach in u-Segment3D involving left-to-right, rasterization of floating-point coordinates onto a discrete image pixel grid, building a count of the number of points within each pixel, approximate Gaussian kernel density estimation using a Gaussian filter of σ, binary thresholding on the mean density and subsequent connected component analysis to identify distinct spatial clusters. **b)** Verification of algorithmic stability by application to foreground (x,y) coordinates after propagation of 50 iterations with gradient descent. 2D gradients are computed using the 2D geodesic centroid point source distance transform. (i) Schematic of

the experimental setup for a 2D cell segmentation with densely touching cells (top panel). (ii) The point density, connected component labelling at current (x^t,y^t) coordinates and labels mapped back to initial ($x^{t=0}$,$y^{t=0}$) coordinates (top-to-bottom) after t=0, 10, 20, 30, 40, 50 iterations of gradient descent (left-to-right). (iii) Plot of the number (#) of unique cells predicted (left, black-colored y-axis and line) and number of matched cells with reference cell shapes (right, green-colored y-axis and line) with iteration number, *t* (top). Dashed black horizontal line indicates the true cell number. Plot of F1 score of matching with reference cells (left, black-colored y-axis and line) and the mean intersection-of-union (IoU) of matched cells with reference (right, green-colored y-axis and line) with iteration number, *t* (bottom). **c)** Reference 2D cells of elongated touching bacteria (left), identified unique cells by spatial connected component at gradient descent propagated coordinates after t = 0, 5, 10, 15, 20 iterations (middle), and plot of F1 score matching with reference cells (left, black-colored y-axis and line) and the mean intersection-of-union (IoU) of matched cells with reference (right, green-colored y-axis and line) with iteration #, *t*. **d)** Image-based connected component labeling applied to recover a doughnut (region 1) surrounding a circle (region 2) after 50 iterations of gradient descent. **e)** Comparison of segmentations of an isolated noisy single cell[16] between Cellpose 3D and u-Segment3D based gradient descent and connected component labeling. Left-to-right: 3D rendering of raw volume, 3D segmentation using Cellpose's gradient descent and density-based clustering, and using u-Segment3D's gradient descent and image-based connected component clustering (top row) with respective mid x-y slice (bottom row). **f)** Comparison of segmentations of multiple cells with elongated morphologies[16] between Cellpose 3D and u-Segment3D based gradient descent and connected component labeling. Left-to-right: 3D rendering of raw volume, 3D segmentation using Cellpose's gradient descent and density-based clustering, 3D segmentation using u-Segment3D's gradient descent and image-based connected component clustering (top row) with respective mid x-y slice (bottom row). Black arrowheads highlight examples of fractured single cells due to unstable spatial clustering in Cellpose. Inset: zoom-in on two fractured cells.

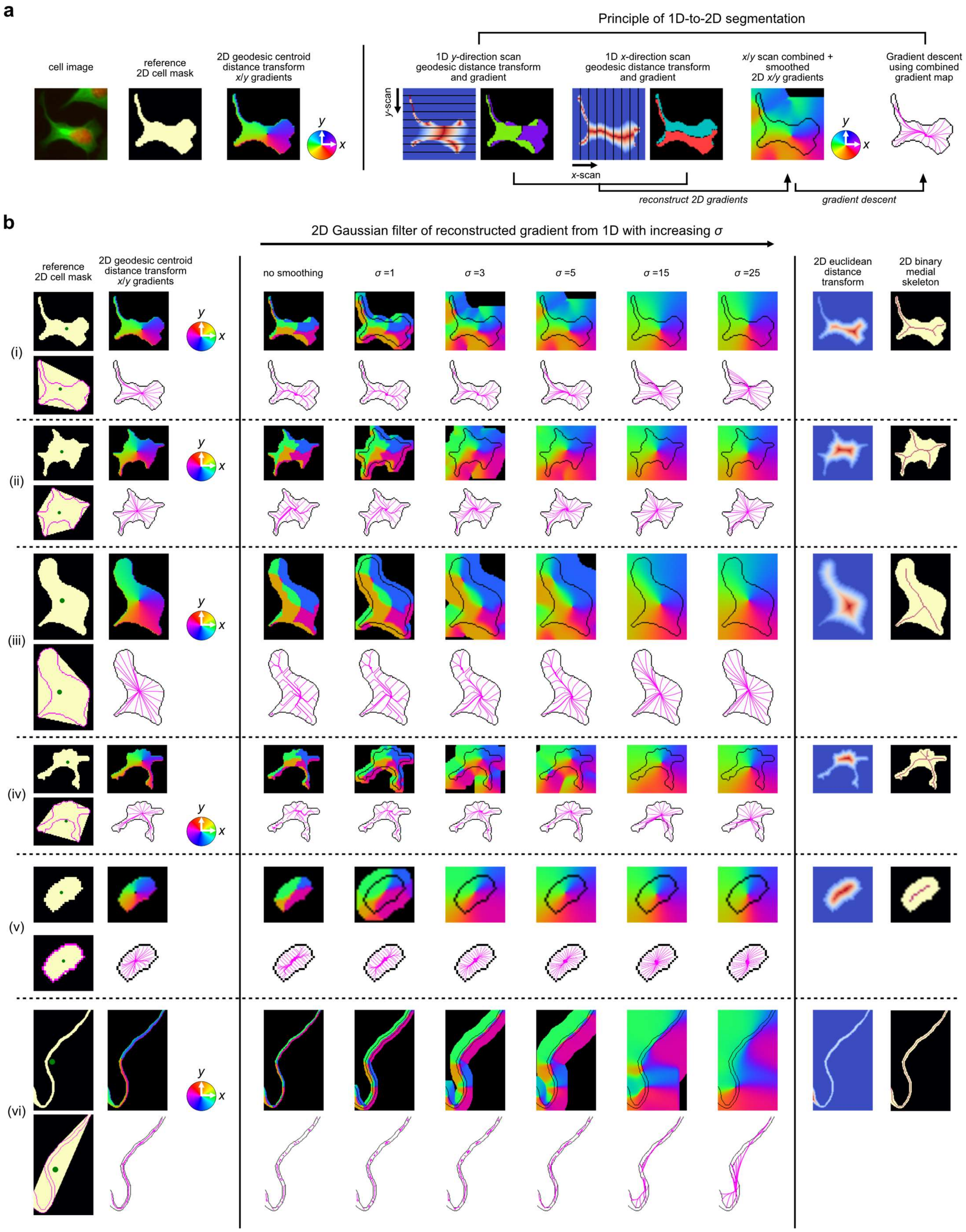

Supplementary Figure 5. 1D-to-2D reconstruction of single cells from ideal 1D segmented stacks. a) Schematic of the workflow to investigate 1D-to-2D segmentation. Reference cell shape and ideal 2D geodesic distance transform to reconstruct (left). Reconstruction of 2D gradient field from computed 1D gradients (right). Left-to-right: Determination of the y-direction and x-direction gradient by computing the distance transform of each pixel in 1D slices to the respective slice centroid and taking the gradient; combining the x- and y-gradient into a 2D gradient field and smoothing with a Gaussian σ; performing gradient descent on the smoothened 2D gradients to propagate all interior points to a unique centroid (magenta line trajectories). **b)**

Gradient descent behavior using Gaussian filter of increasing σ on the initial 2D gradients reconstructed for 6 examples of individual cells with diverse morphologies. (i-iii), examples of approximately star-convex shapes; (iv) example of a cell with branches of lengths comparable to cell body; (v) example of convex shaped cell; (vi) example of a thin vessel-like shaped cell. Left column, montage of 4 images depicting reference cell shape and its convex hull image with green point representing the centroid coordinate and the cell's exact 2D geodesic centroid distance transform with associated gradient descent trajectory to reconstruct. Middle column, observed gradient descent trajectory (magenta line) when the reconstructed 2D gradients are smoothed with an isotropic Gaussian filter of increasing σ. Right column, comparison of the gradient descent trajectory with the implicit medial skeleton specified by the 2D Euclidean distance transform and explicit medial skeleton from morphological operations.

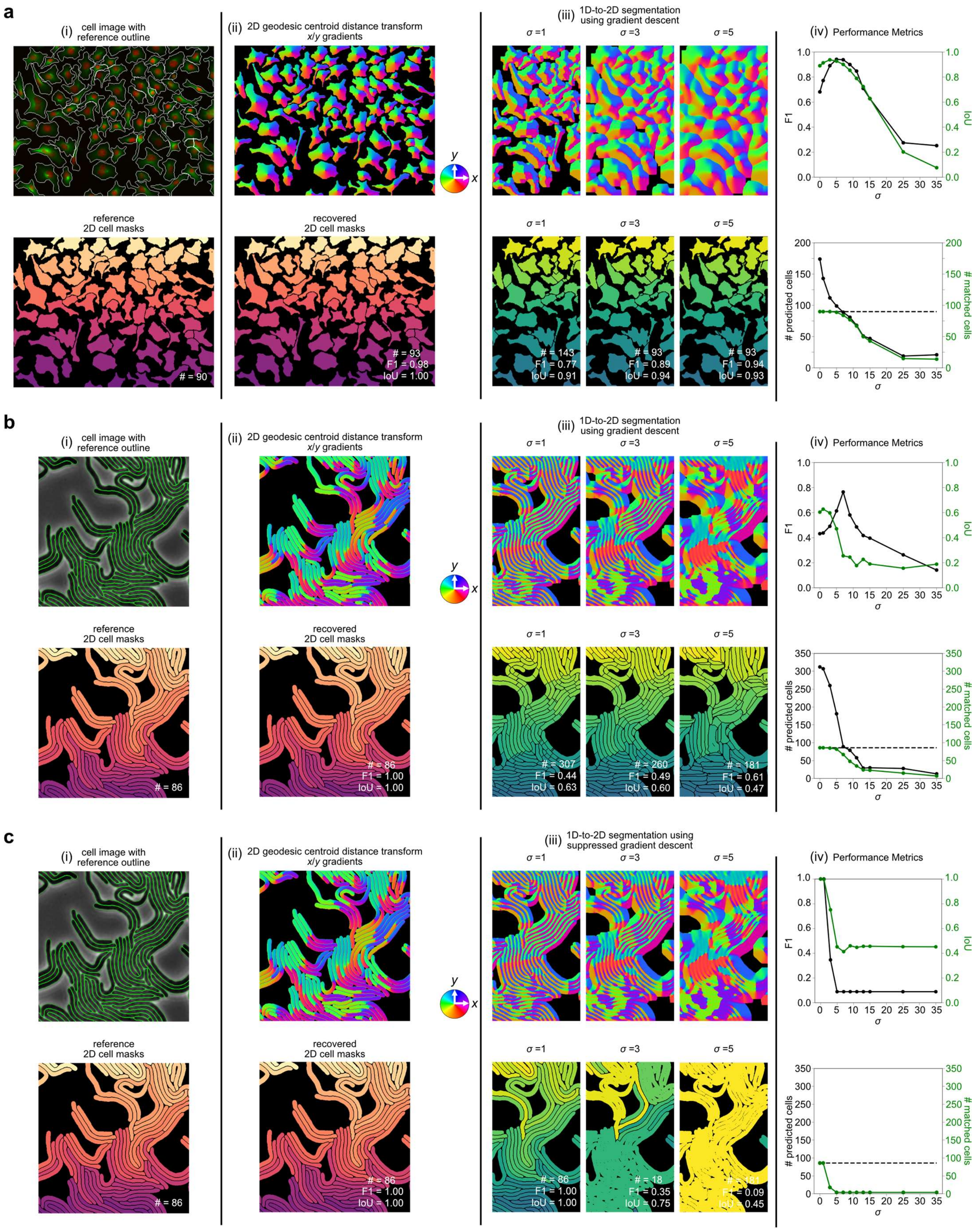

Supplementary Figure 6. 1D-to-2D reconstruction for densely packed cell shapes. a) Representative example of a dense 2D cell culture with diverse morphologies with (i) reference cell boundaries overlaid and delineated in white (top) and uniquely colored cell masks (bottom). (ii) Exact unit-normalized 2D gradients of the geodesic centroid distance transform colored by direction (top) and the recovered cell masks using connected component labeling after 100 iterations of gradient descent with step-size of one pixel. (iii) Reconstructed 2D gradient from 1D after Gaussian filtering with increasing σ left-to-right (top) and corresponding recovered cell masks using connected component labeling after 100 iterations of gradient

descent. (iv) Performance of the 1D-to-2D reconstruction with increasing σ. F1 score of matching with reference cells (left, black-colored y-axis and line) and mean intersection-of-union (IoU) of matched cells with reference (right, green-colored y-axis and line) (top). Number (#) of distinct predicted cells (left, black-colored y-axis and line) and # of those that could be matched with reference cells (right, green-colored y-axis and line) (bottom). Dashed black horizontal line indicates the true cell number. **b)** Representative example of a dense 2D cell culture with highly elongated, vessel-like cells. Panels (i)-(iv) as in a). **c)** Same example image and panels (i)-(iv) as in b) but using suppressed gradient descent where the step-size $\eta = \frac{1}{1+\tau \cdot t}$, is attenuated with increasing gradient descent iteration number, t and $\tau = 1$ (Methods).

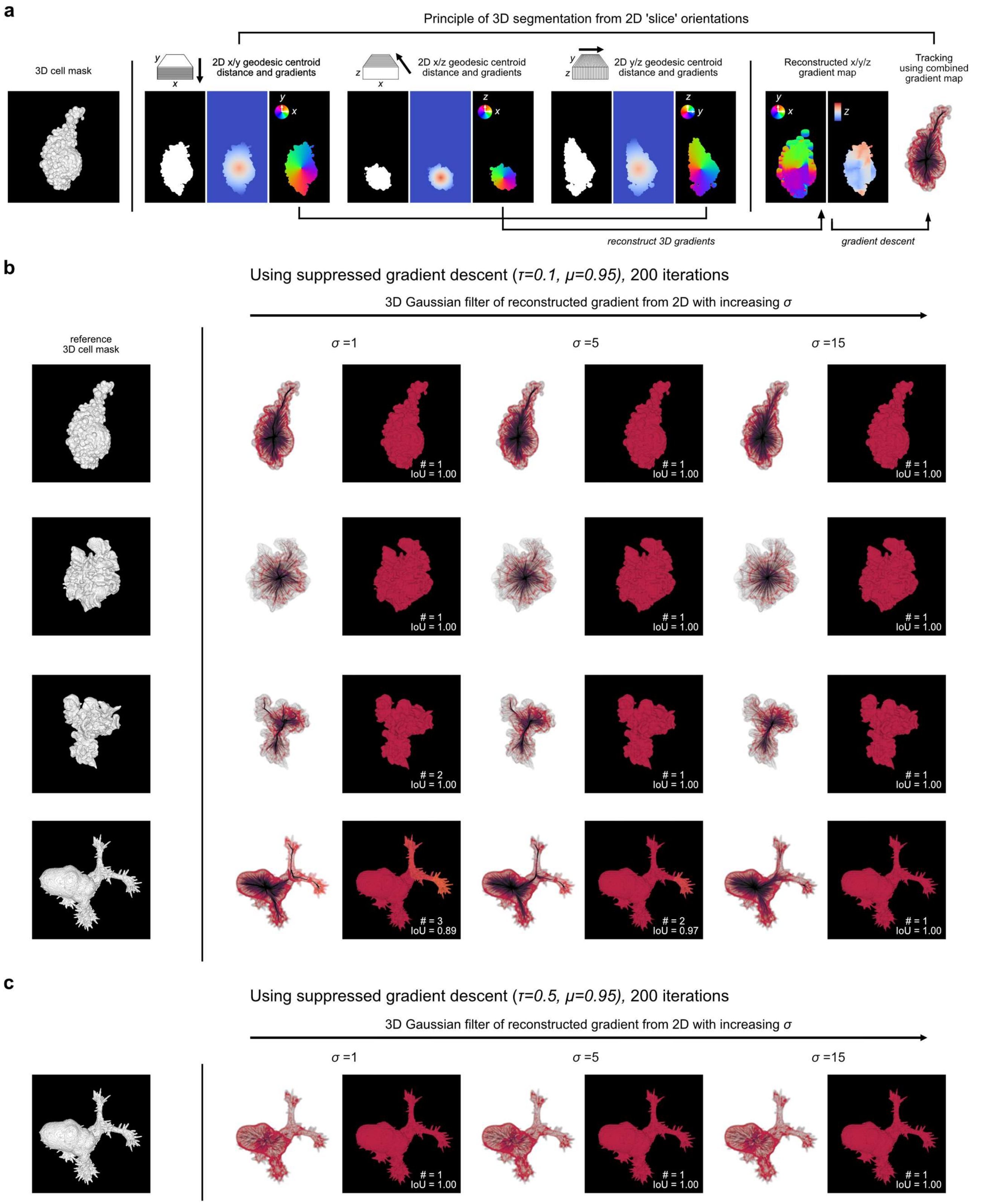

Supplementary Figure 7. 2D-to-3D reconstruction of single cells from ideal 2D segmented stacks. a) Illustration of the reconstruction experiment given the 3D shape of a single cell (left), by generating 2D gradients slice-by-slice in x-y, x-z, y-z views, treating each disconnected spatial component as a unique 2D cell (middle) and performing gradient descent on the reconstructed 3D x/y/z gradient followed by connected component labeling on the final advected 3D coordinates (right). **b)** Reconstruction of cells with blebs (1st row), lamellipodia (2nd, 3rd rows) and filopodia (4th row). In each row, left-to-right: reference binary 3D cell shape, the 3D gradient descent trajectory (left) and reconstructed 3D shape (right) for Gaussian filtering of the 3D reconstructed gradients with $\sigma = 1,5,15$, using suppressed gradient descent ($\tau = 0.1$) with momentum

($\mu = 0.95$). **c).** Reconstruction of the same cells with filopodia in b) for post- Gaussian filtering with $\sigma = 1,5,15$ (left-to-right), using suppressed gradient descent with greater decay ($\tau = 0.5$) with momentum ($\mu = 0.95$).

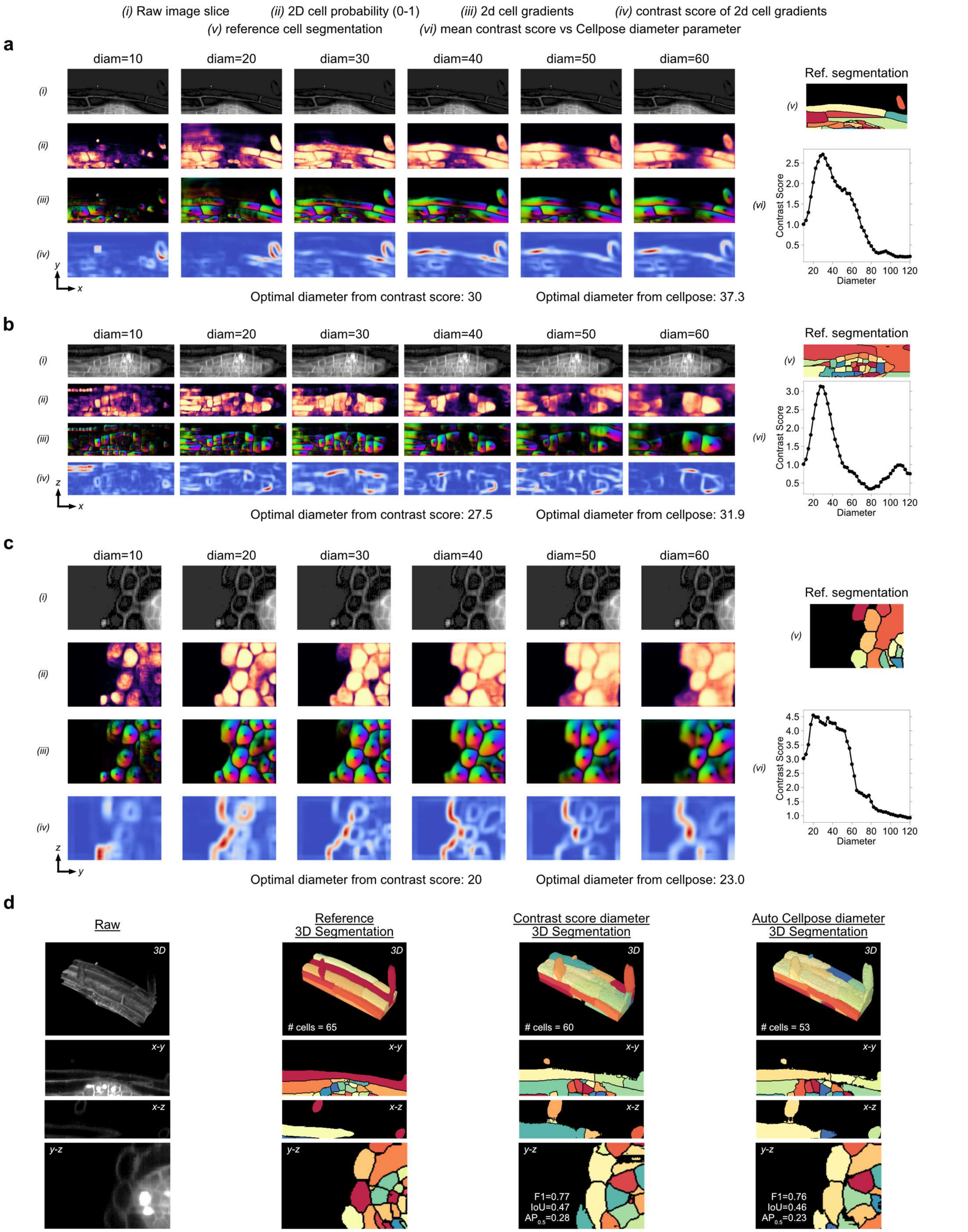

Supplementary Figure 8. The diameter parameter in pretrained Cellpose models must be individually set in orthogonal views. a) Cellpose 'cyto2' model outputs and per-pixel u-Segment3D contrast score on the most in-focus (Methods) 2D x-y slice of a Lateral Root Primordia image as diameter (diam) is increased. (i) Raw input image slice, the same for all values of diameter. (ii) Normalized (0-1) Cellpose 2D pixel probability map colored black=0 to yellow=1. (iii) Unit-normalized Cellpose 2D predicted gradients colored by direction. (iv) Contrast score of predicted Cellpose 2D gradient (Methods, Extended Data Figure 10a). (v) Reference cell segmentation for the raw 2D input. (vi) Mean contrast score averaged over the image for each

value of diam. The diameter with maximum contrast score is taken as the optimal diameter by u-Segment3D. **b)** Same as in a) for the most in-focus 2D x-z slice and **c)** the most in-focus 2D y-z slice. **d)** From left-to-right, 3D renderings of the raw 3D volume, reference 3D segmentation, u-Segment3D segmentation using the direct method and diameter in each orthoview set by contrast score or by the Cellpose 'cyto2' model (top), and corresponding mid-plane orthoslices in all three views (below). All results are representative of the n=2 images in the validation split of the Lateral Root Primordia dataset.

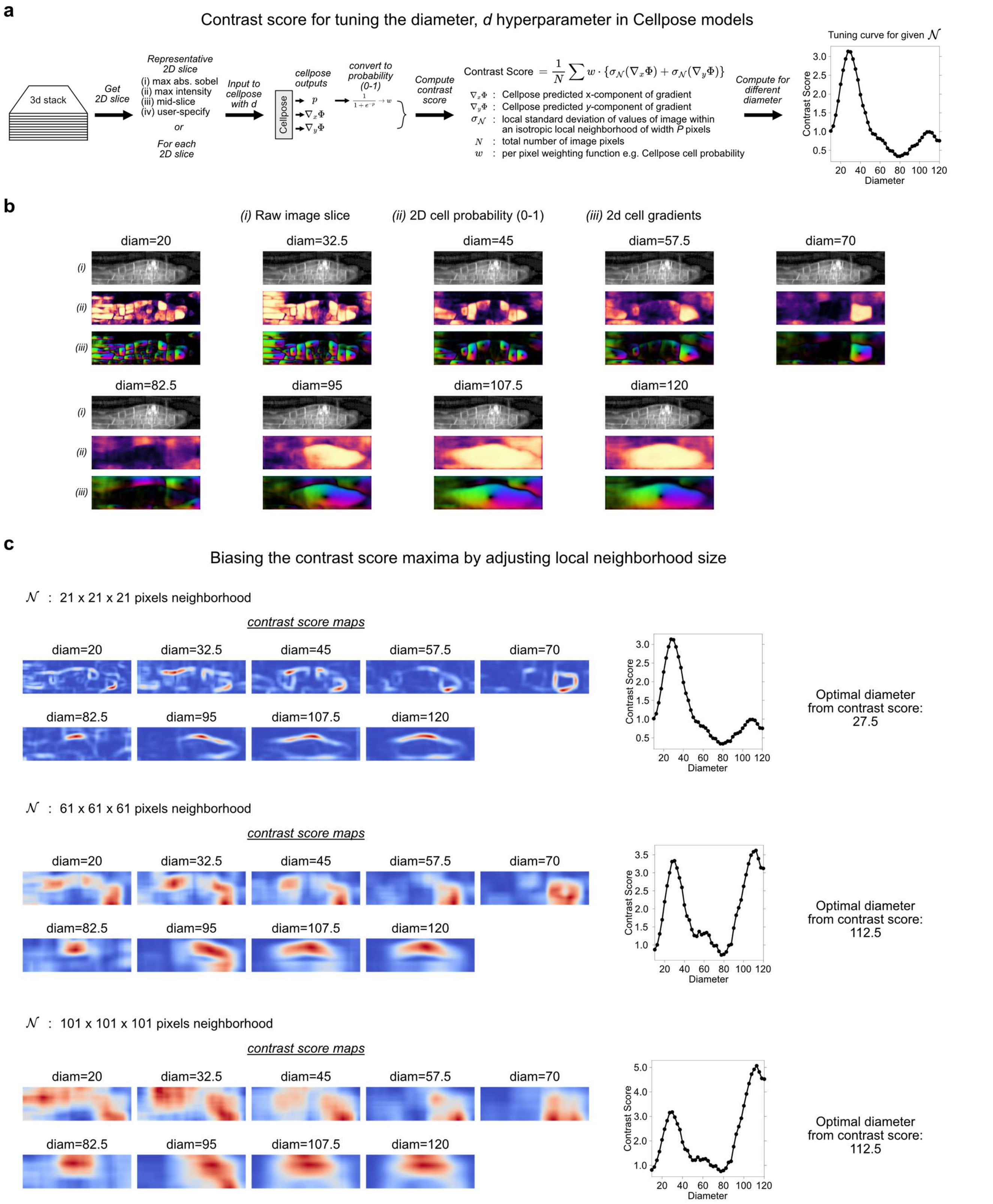

Supplementary Figure 9. Semi-automatic determination of the diameter parameter in pretrained Cellpose models using local variance. a) Computational workflow and definition of a contrast score function using a local neighborhood of width P pixels to evaluate 2D Cellpose outputs when the diameter parameter is set to d. **b)** (i) Raw input image slice, (ii) normalized (0-1) Cellpose 2D pixel probability map colored black=0 to yellow=1, and (iii) unit-normalized Cellpose 2D predicted gradients colored by direction for 9 equisampled diameters in the range $d = [20, 120]$. **c)** Contrast score maps (colored blue-to-red for low-to-high values) for the same d as in b) (left) and the resulting contrast score function and optimal diameter inferred (right), using a neighborhood of width $P = 21, 61, 101$ pixels (top-to-bottom). All results are representative of the n=2 images in the validation split of the Lateral Root Primordia dataset.

Supplementary References

1 Tagliasacchi, A., Delame, T., Spagnuolo, M., Amenta, N. & Telea, A. in *Eurographics.* 573-597 (2016).

2 Lam, L., Lee, S.-W. & Suen, C. Y. Thinning methodologies-a comprehensive survey. *IEEE Transactions on Pattern Analysis & Machine Intelligence* **14**, 869-885 (1992).

3 Stringer, C., Wang, T., Michaelos, M. & Pachitariu, M. Cellpose: a generalist algorithm for cellular segmentation. *Nature methods* **18**, 100-106 (2021).

4 Sethian, J. A. A fast marching level set method for monotonically advancing fronts. *proceedings of the National Academy of Sciences* **93**, 1591-1595 (1996).

5 Cutler, K. J. *et al.* Omnipose: a high-precision morphology-independent solution for bacterial cell segmentation. *Nature methods* **19**, 1438-1448 (2022).

6 Schmidt, U., Weigert, M., Broaddus, C. & Myers, G. in *Medical Image Computing and Computer Assisted Intervention–MICCAI 2018: 21st International Conference, Granada, Spain, September 16-20, 2018, Proceedings, Part II 11.* 265-273 (Springer).

7 Preibisch, S., Rohlfing, T., Hasak, M. P. & Tomancak, P. in *Medical Imaging 2008: Image Processing.* 128-135 (SPIE).

8 Preibisch, S. *et al.* Efficient Bayesian-based multiview deconvolution. *Nature methods* **11**, 645-648 (2014).

9 van Ineveld, R. L. *et al.* Revealing the spatio-phenotypic patterning of cells in healthy and tumor tissues with mLSR-3D and STAPL-3D. *Nature biotechnology* **39**, 1239-1245 (2021).

10 Stringer, C. & Pachitariu, M. Cellpose3: one-click image restoration for improved cellular segmentation. *Nature Methods*, 1-8 (2025).

11 Driscoll, M. K. *et al.* Robust and automated detection of subcellular morphological motifs in 3D microscopy images. *Nat Methods* **16**, 1037-1044, doi:10.1038/s41592-019-0539-z (2019).

12 Zhou, F. Y. *et al.* Surface-guided computing to analyze subcellular morphology and membrane-associated signals in 3D. *bioRxiv*, 2023.2004. 2012.536640 (2023).

13 Wolny, A. *et al.* Accurate and versatile 3D segmentation of plant tissues at cellular resolution. *Elife* **9**, e57613 (2020).

14 Lalit, M., Tomancak, P. & Jug, F. Embedseg: Embedding-based instance segmentation for biomedical microscopy data. *Medical image analysis* **81**, 102523 (2022).

15 Nanes, B. A. *et al.* Shifts in keratin isoform expression activate motility signals during wound healing. *Developmental cell* **59**, 2759-2771. e2711 (2024).

16 Maška, M. *et al.* The Cell Tracking Challenge: 10 years of objective benchmarking. *Nature Methods* **20**, 1010-1020 (2023).

17 Heemskerk, I. & Streichan, S. J. Tissue cartography: compressing bio-image data by dimensional reduction. *Nature methods* **12**, 1139-1142 (2015).
