# Supplementary material for: Universal consensus 3D segmentation of cells from 2D segmented stacks
Source: Nat Methods. 2025 Nov 11;22(11):2386–99. doi: 10.1038/s41592-025-02887-w (PMC12615268; doi:10.1038/s41592-025-02887-w)
Supplement: Supplementary file 2 — Reporting Summary [file 41592_2025_2887_MOESM2_ESM.pdf]

nature portfolio

Corresponding author(s): Gaudenz Danuser, Felix Zhou

Last updated by author(s): Jun 23, 2025

Reporting Summary

Nature Portfolio wishes to improve the reproducibility of the work that we publish. This form provides structure for consistency and transparency in reporting. For further information on Nature Portfolio policies, see our Editorial Policies and the Editorial Policy Checklist.

Statistics

For all statistical analyses, confirm that the following items are present in the figure legend, table legend, main text, or Methods section.

n/a	Confirmed	
☐	☒	The exact sample size (*n*) for each experimental group/condition, given as a discrete number and unit of measurement
☐	☒	A statement on whether measurements were taken from distinct samples or whether the same sample was measured repeatedly
☒	☐	The statistical test(s) used AND whether they are one- or two-sided<br>*Only common tests should be described solely by name; describe more complex techniques in the Methods section.*
☒	☐	A description of all covariates tested
☒	☐	A description of any assumptions or corrections, such as tests of normality and adjustment for multiple comparisons
☐	☒	A full description of the statistical parameters including central tendency (e.g. means) or other basic estimates (e.g. regression coefficient) AND variation (e.g. standard deviation) or associated estimates of uncertainty (e.g. confidence intervals)
☒	☐	For null hypothesis testing, the test statistic (e.g. *F*, *t*, *r*) with confidence intervals, effect sizes, degrees of freedom and *P* value noted<br>*Give P values as exact values whenever suitable.*
☒	☐	For Bayesian analysis, information on the choice of priors and Markov chain Monte Carlo settings
☒	☐	For hierarchical and complex designs, identification of the appropriate level for tests and full reporting of outcomes
☐	☒	Estimates of effect sizes (e.g. Cohen's *d*, Pearson's *r*), indicating how they were calculated

Our web collection on statistics for biologists contains articles on many of the points above.

Software and code

Policy information about availability of computer code

Data collection	Original microscopy (single cells(ruffles)) was acquired with a home-built light-sheet fluorescence microscope controlled by the software developed by Coleman technologies. It uses a 64-bit version of LabView 2016 equipped with the LabView Run-Time Engine, Vision Development Module and Vision Run-Time Module (National Instruments); navigate (https://github.com/TheDeanLab/navigate) developed by the Dean Lab (for cleared tissue); Zeiss software (for T cell co-culture); custom multi-scale light-sheet microscope with axially-swept light-sheet microscopy and controlled by custom Python software (https://github.com/DaetwylerStephan/self_driving_multiscale_control) (for Zebrafish vasculature)
Data analysis	u-Segment3D is available at https://github.com/DanuserLab/u-segment3D. Unwrapping of drosophila surface was performed with u-Unwrap3D available at https://github.com/DanuserLab/u-unwrap3D. Experiments in the paper except for Fig. 3, and its associated Extended Data Fig. 6,7 were performed prior to publication of Cellpose3 and uses Cellpose version 2.3.dev7+g03e02bc. The same conclusion does however apply to Cellpose 3 (the latest Cellpose version), which would be the version now installed by the u-Segment3D package. Fig. 3, and its associated Extended Data Fig. 6,7 used Cellpose3 (version 3.0.8). Omnipose used version 0.3.5.dev10+ge22262b. EmbedSeg used version 0.2.5. StarDist used version 0.9.1. PlantSeg used version 1.6.0. CellStitch used version 1.0.0. 3DCellComposer used version 1.2.1.

For manuscripts utilizing custom algorithms or software that are central to the research but not yet described in published literature, software must be made available to editors and reviewers. We strongly encourage code deposition in a community repository (e.g. GitHub). See the Nature Portfolio guidelines for submitting code & software for further information.

Data

Policy information about availability of data

All manuscripts must include a data availability statement. This statement should provide the following information, where applicable:

- Accession codes, unique identifiers, or web links for publicly available datasets
- A description of any restrictions on data availability
- For clinical datasets or third party data, please ensure that the statement adheres to our policy

All data used in this study are publicly available. The already published segmentation datasets used for benchmarking and example demonstration are available from their sources as documented in Suppl. Table 2 and in the Dataset section of Methods. The original microscopy data generated for this paper are made available in a Zenodo repository, (https://doi.org/10.5281/zenodo.15692302)

Human research participants

Policy information about studies involving human research participants and Sex and Gender in Research.

Reporting on sex and gender	Samples were used for the purposes of demonstrating cell segmentation and not for obtaining biological insight. We did not consider sex and gender.
Population characteristics	Samples were used for the purposes of demonstrating cell segmentation and not for obtaining biological insight. No population characteristics or other covariates were considered.
Recruitment	Samples were used to demonstrate cell segmentation and not for obtaining biological insight. No specific recruitment criteria were used. T cells were obtained from blood leukocyte cones purchased from NHS Blood and Transplant, John Radcliffe Hospital, Oxford, UK. Blood cones were used under the ethical guidelines of the NHS Blood and Transplant. The Non-Clinical Issue division of National Health Service approved the use of blood leukocyte cones at the University of Oxford (REC 11/H0711/11).
Ethics oversight	T cells were obtained from blood leukocyte cones purchased from NHS Blood and Transplant, John Radcliffe Hospital, Oxford, UK. Blood cones were used under the ethical guidelines of the NHS Blood and Transplant. The Non-Clinical Issue division of National Health Service approved the use of blood leukocyte cones at the University of Oxford (REC 11/H0711/11).

Note that full information on the approval of the study protocol must also be provided in the manuscript.

Field-specific reporting

Please select the one below that is the best fit for your research. If you are not sure, read the appropriate sections before making your selection.

☒ Life sciences ☐ Behavioural & social sciences ☐ Ecological, evolutionary & environmental sciences

For a reference copy of the document with all sections, see nature.com/documents/nr-reporting-summary-flat.pdf

Life sciences study design

All studies must disclose on these points even when the disclosure is negative.

Sample size	For benchmarking u-Segment3D we used all available data in the public datasets. Additional datasets were selected to demonstrate further unique u-Segment3D features, not to derive biological conclusions or evaluate effect size. We use a pretrained Cellpose model and the rest of the calculations in u-Segment3D is deterministic therefore they are all n=1 demonstrations.
Data exclusions	We used all applicable data from public datasets for benchmarking experiments but had to exclude one image from DeepVesselNet which did not have both image and reference segmentation.
Replication	u-Segment3D is comprehensively benchmarked on >70,000 cells from 11 public datasets comprising diverse cell morphologies: convex, concave, branching and networks and from different cell densities: single cells, cell aggregates and tissue. All attempts at replication were successful. Demonstration of u-Segment3D on additional datasets used a pretrained Cellpose model and u-Segment3D algorithms are all deterministic, therefore results are by definition all replicable, given the same installed Cellpose version.
Randomization	Per standard segmentation benchmarking, we used the designated train/test/val splits provided by the public datasets. In Fig. 3 we generated train/test/val splits for datasets that did not have these splits, by random sampling. We follow the random sampling procedure detailed by EmbedSeg paper and used the same splits for all other algorithms to be fair. Additional experiments only seek to demonstrate features of u-Segment3D therefore no randomization was required (all computations are deterministics), n=1 were used per example.
Blinding	Blinding is not applicable for segmentation where all dataset is used, and there are no confounding covariates that would bias the result. Per standard segmentation benchmarking, we used the designated train/test/val splits provided by the public datasets. In Fig. 3 we generated train/test/val splits for datasets that did not have these splits, by random sampling. We follow the random sampling procedure detailed by

EmbedSeg paper and used the same splits for all other algorithms to be fair. Additional experiments only seek to demonstrate features of u-Segment3D therefore no randomization was required (all computations are deterministics), n=1 were used per example.

Reporting for specific materials, systems and methods

We require information from authors about some types of materials, experimental systems and methods used in many studies. Here, indicate whether each material, system or method listed is relevant to your study. If you are not sure if a list item applies to your research, read the appropriate section before selecting a response.

Materials & experimental systems

n/a	Involved in the study
☐	☒ Antibodies
☐	☒ Eukaryotic cell lines
☒	☐ Palaeontology and archaeology
☐	☒ Animals and other organisms
☒	☐ Clinical data
☒	☐ Dual use research of concern

Methods

n/a	Involved in the study
☒	☐ ChIP-seq
☒	☐ Flow cytometry
☒	☐ MRI-based neuroimaging

Antibodies

Antibodies used	Primary antibody: Anti-Green Fluorescent Protein (GFP) chicken polyclonal primary antibody, Supplier Name: Aves Labs, Catalog Number: # GFP-1020<br><br>Secondary antibody: Alexa Fluor 488 AffiniPure F(ab')$_2$ Fragment Donkey Anti-Chicken IgY (IgG) (H+L), polyclonal, Supplier Name: Fisher Scientific, Catalog Number: # NC0456003
Validation	Anti-Green Fluorescent Protein (GFP) chicken polyclonal primary antibody were analyzed by western blot analysis (1:5000 dilution) and immunhistochemistry (1:500 dilution) using transgenic mice expressing the GFP gene product. Western blots were performed using BiokHen (Aves Labs) as the blocking reagent, and HRP-labeled goat anti-chicken antibodies (Aves Labs, Cat. #H-1004) as the detection reagent. Immunohistochemistry used tetramethyl rhodamine-labeled anti-chicken IgY.

Eukaryotic cell lines

Policy information about cell lines and Sex and Gender in Research

Cell line source(s)	MV3 cells were gifted by Peter Friedl (MD Anderson Cancer Center, Houston TX), whose lab established the line (DOI:10.1002/ijc.2910480116). We are unaware of a commercial source for this cell line.<br><br>Ker-CT cells (ATCC CRL-4048) were gifted by Dr. Jerry Shay (UT Southwestern Medical Center).<br><br>Blasted human CD8+ T cells, were produced by activating naïve T cells isolated from PBMCs.<br><br>COR-L23 cells were from Millipore Sigma<br><br>YUMM 1.7 cells were from ATCC.
Authentication	MV3 cells were authenticated using GenePrint 10 System from Promega.<br><br>Ker-CT cells were not authenticated.<br><br>Naïve T cells were checked using flow cytometry e.g. (CD3+CD45R0-CCR7+)<br><br>COR-L23 cells were not authenticated.<br><br>YUMM 1.7 cells were not authenticated.
Mycoplasma contamination	Cell lines were periodically tested and negative by PCR for mycoplasma contamination
Commonly misidentified lines<br>(See ICLAC register)	No misidentified cell lines were used in this study

Animals and other research organisms

Policy information about studies involving animals; ARRIVE guidelines recommended for reporting animal research, and Sex and Gender in Research

Laboratory animals	Danio Rerio (Zebrafish, strains: Tg(kdrl:Hsa.HRAS-mcherry) in a casper background.

Laboratory animals	Age: 3-5 dpf, therefore the sex of the organism was not yet determined.<br><br>Mouse strains: NSG (https://www.jax.org/strain/005557, doi: 10.1038/s41586-020-2623-z)<br>Age: Cancer cell injection around 6 weeks. Tumors take about a month to grow.<br>Housing Conditions: Mice were housed at the Animal Resource Center at the University of Texas Southwestern Medical Center in Association for Assessment and Accreditation of Laboratory Animal Care International - accredited, specific pathogen-free animal care facilities under a 12 hours: 12 hours light:dark cycle with a temperature of 18c to 24c and humidity of 35% to 60%.
Wild animals	No wild animals were used.
Reporting on sex	The imaging experiments were conducted using both male and female animals. The purpose of the paper is to achieve cell segmentation irrespective of gender.
Field-collected samples	No field-collected samples were used.
Ethics oversight	All zebrafish husbandry and experiments described here have been approved and conducted under the oversight of the Institutional Animal Care and Use Committee (IACUC) at UT Southwestern under protocol number 101805.<br><br>All mouse experiments complied with all relevant ethical regulations and were performed according to protocols approved by the Institutional Animal Care and Use Committee (IACUC) at the University of Texas Southwestern Medical Center (protocol 2016-101360).

Note that full information on the approval of the study protocol must also be provided in the manuscript.
